# Supplementary material for: Direct factor Xa inhibitors and the risk of cancer and cancer mortality: A Danish population-based cohort study
Source: PLoS Med. 2024 Jul 1;21(7):e1004400. doi: 10.1371/journal.pmed.1004400 (PMC11251598; doi:10.1371/journal.pmed.1004400)
Supplement: S1 Table — ICD-8, 8th revision of the International Statistical Classification of Diseases and Related Health Problems; ICD-10, 10th revision of the International Statistical Classification of Diseases and Related Health Problems. (DOCX) [file pmed.1004400.s002.docx]

**S1 Table. Index variable, covariates, and non-cancer-related outcomes from the DNPR**

| **Diagnosis** | **ICD-8 code** | **ICD-10 code** |
| --- | --- | --- |
| Atrial fibrillation and flutter | 427.92 | I48 |
| Bleeding in the upper  gastrointestinal tract | 530.98, 531.90, 531.92, 531.95, 532.90, 533.90, 534.90, 535.01, 456.01 | I85.0, K25.0, K25.2, K25.4, K25.6, K26.0, K26.2, K26.4, K26.6, K27.0, K27.2, K27.4, K27.6, K28.0, K28.2, K28.4, K28.6, K29.01, K29.21, K29.31, K29.41, K29.51, K29.61, K29.71, K29.81, K29.91 |
| Bleeding in the lower  gastrointestinal tract | 569.15 | K62.5, K92.0–K92.2 |
| Myocardial infarction |  |  |
| Congestive heart failure | 42709, 42710, 42711, 42719, 42899, 78249 | I500, I501, I502, I503, I508, I509, I110, I130, I132, I420, I426, I427, I428, I429 |
| Ischemic stroke | 433–434 | I63 |
| Chronic obstructive pulmonary  disease | 491; 492 | J40–J44 |
| Liver disease | 070.00; 070.02; 070.04; 070.06; 070.08; 456.00–456.09; 571; 573.00; 573.01; 573.04 | B15.0; B16.0; B16.2; B18; B19.0; K70.0–K70.3; K70.4; K70.9; K71–K74; K76.0; K76.6; I85 |
| Renal disease | 403; 404; 580–583; 584; 590.09; 593.19; 753.10–753.19; 792 | I12; I13; N00–N05; N07; N11; N14; N17–N19; Q61 |
| Inflammatory bowel disease | 563 | K50–K52 |
| Pancreatitis | 577.0; 577.1; 577.10 | K85; K86.0; K86.1 |
| Gallstones | 574 | K80 |
| Diabetes mellitus | 249.00, 249.06, 249.07, 249.09, 250.00, 250.06, 250.07, 250.09 | E10–E13;  O24 (except O24.4), G63.2, H36.0, N08.3 |
| Hypertension | 400.09–404.99, 410.09, 411.09, 412.09, 413.09, 414.09, 435.09, 437.00–437.09, 438.09 | I10.0, I10.9, I11.0, I11.9, I12.0,  I12.9, I13.0–I13.9, I15.0–I15.9 |
| Rheumatoid arthritis | 712 | M05; M06 |
| Alcohol dependency | 291; 303; 979; 577.10; 571.09; 571.10 | F10; E24.4; G31.2; G62.1; G72.1; I42.6; K29.2; K70; K86.0; O35.4; Y57.3; Z50.2; Z71.4; Z72.1 |
| Obesity and obesity-related disorders | 277 | E65–E68 |

**Abbreviations:** ICD-8, 8^th^ revision of the International Statistical Classification of Diseases and Related Health Problems; ICD-10, 10^th^ revision of the International Statistical Classification of Diseases and Related Health Problems
