# Supplementary material for: Direct factor Xa inhibitors and the risk of cancer and cancer mortality: A Danish population-based cohort study
Source: PLoS Med. 2024 Jul 1;21(7):e1004400. doi: 10.1371/journal.pmed.1004400 (PMC11251598; doi:10.1371/journal.pmed.1004400)
Supplement: S2 Table — *Used to define diagnosis. ATC, Anatomical Therapeutic Chemical; NSAIDs, nonsteroidal anti-inflammatory drugs. (DOCX) [file pmed.1004400.s003.docx]

**S2 Table. Drugs and ATC codes**

| **Drug** | **ATC code** |
| --- | --- |
| *Direct thrombin inhibitors* |  |
| Dabigatran | B01AE07 |
| *Direct factor Xa inhibitors* |  |
| Rivaroxaban | B01AF01; B01AX06 |
| Apixaban | B01AF02 |
| Edoxaban | B01AF03 |
| Platelet aggregation inhibitors | B01AC |
| Antihypertensive agents* | C02A, C02B, C02C, C02DA, C02L, C03A, C03B, C03D, C03E, C03X, C07C, C07D, C08G, C09BA, C09DA, C09XA52, C02DB, C02DD, C02DG, C04, C05, C07 |
| Diabetes medication* | A10 |
| Drugs for alcohol dependency* | N07BB01–3 |
| Lipid lowering drugs | C10A |
| Glucocorticoids | H02 |
| NSAIDs | M01A |
| Strong analgesics | N02A |
| Antidepressants | N06A |

*Used to define diagnosis

**Abbreviations:** ATC, Anatomical Therapeutic Chemical; NSAIDs, non-steroidal anti-inflammatory drugs
