# Supplementary material for: Direct factor Xa inhibitors and the risk of cancer and cancer mortality: A Danish population-based cohort study
Source: PLoS Med. 2024 Jul 1;21(7):e1004400. doi: 10.1371/journal.pmed.1004400 (PMC11251598; doi:10.1371/journal.pmed.1004400)
Supplement: S3 Table — ICD-10, 10th revision of the International Statistical Classification of Diseases and Related Health Problems. (DOCX) [file pmed.1004400.s004.docx]

**S3 Table. Table of cancer diagnosis and cancer groups**

| **Cancers** | **ICD-10 codes** |
| --- | --- |
| *All cancers* | C00–C96 (excluding C44 and C4A) |
|  |  |
| *Obesity-related cancers* |  |
| Esophagus | C15 |
| Pancreas | C25 |
| Colon including the rectosigmoid junction | C18–C19 |
| Rectum | C20 |
| Breast, postmenopausal (≥60 years) | C50 |
| Uterus | C54–C55 |
| Kidney | C64 |
| Gallbladder and bile ducts | C23–C24 |
| Thyroid gland | C73 |
| *Smoking- and alcohol-related cancers* |  |
| Lip | C00 |
| Tongue | C01–02 |
| Mouth | C03–06 |
| Tonsil and pharynx | C09–C13 |
| Other and poorly specified locations in lip, oral cavity, and pharynx | C14 |
| Larynx | C32 |
| Other and poorly specified locations in airways and respiratory organs | C39 |
| Stomach | C16 |
| Small intestine | C17 |
| Liver, including intrahepatic bile ducts | C22 |
| Lung, bronchus, and trachea | C33–C34 |
| Renal pelvis | C65 |
| Ureter | C66 |
| Urinary bladder | C67 |
| *Hematological cancers* |  |
| Hodgkin’s lymphoma (included morphologic code 965–966) | C81 |
| Non-Hodgkin’s lymphoma, excluding leukemia and myelomatosis (including morphologic code 959, 967–972) | C82–85 |
| Malignant myeloproliferative disease | C88 |
| Multiple myeloma and other plasma cell neoplasms | C90 |
| Myeloid leukemia | C92 |
| Lymphocytic leukemia | C91 |
| Monocytic leukemia | C93 |
| Other leukemia | C94–95 |
| Other and unspecified cancers of lymphoid, hematopoietic, and related tissues | C96 |
| Metastasis and unspecified cancer in lymph nodes (only when no primary tumor is coded) | C77–79 (only if no primary tumor is coded) |
| *Immune-related cancers* |  |
| Anus and anal canal, excluding malignant melanomas (morphologic code 872–879) and basal cell cancers (morphologic code 809) | C21 |
| Cervix | C53 |
| External female genitalia, excluding basal cell carcinomas (morphological code 809) | C51 |
| Penis, excluding basal cell carcinomas (morphological code 809) | C60 |
| Malignant melanoma, including those located in anus and anal canal (morphological code 872–879) | C43 |
| Non-melanoma skin cancers, excluding basal cell carcinoma (morphologic code 809) | C44 |
| *Cancers of neurological origin* |  |
| Meningioma | C70, D32, D42 |
| Brain, including hypophysis, corpus pineale, and ductus craniopharyngealis | C71, C751–753, D330-D332, D352-D354, D430-D432, D443-D445 |
| Spinal cord, cranial nerves, and other parts of central nervous system | C72, D333-D339, D433-D439 |
| *Hormone-related cancers* |  |
| Prostate | C61 |
| Testicular | C62 |
| Vagina, excluding basal cell carcinomas (morphological code 809) | C52 |
| Breast, premenopausal (<60 years) | C50 |
| Ovary and fallopian tube | C56, C570–574 |
| *All other cancers* |  |
| Salivary glands | C07–08 |
| Other and ill-defined cancers of digestive organs | C26 |
| Nasal cavity, middle ear, and accessory sinuses | C30-C31 |
| Thymus | C37 |
| Heart and mediastinum | C381–383, C388 |
| Pleura, including mesothelioma pleura | C384, C450 |
| Bone and articular cartilage | C40-C41 |
| Kaposi’s sarcoma | C46, B210 |
| Mesothelioma | C45·1–C45·9 |
| Peripheral nerves and autonomic nervous system | C47 |
| Retroperitoneum and peritoneum | C48 |
| Malignant neoplasm of other connective and soft tissue | C49 |
| Placenta | C58 |
| Other and unspecified cancers in female genital organs | C577–579 |
| Other and unspecified cancers in male genital organs, excluding basal cell carcinomas (morphological code 809) | C63 |
| Other and unspecified cancers in urinary organs | C68 |
| Eye and adnexa |  |
| Adrenal gland | C74 |
| Other endocrine structures, excluding hypophysis, corpus pineale, and ductus craniopharyngealis | C750, C754–759 |
| Malignant neoplasm at other, ill-defined, or unspecified sites | C76, C80 |
| Malignant neoplasms at independent (primary) multiple sites | C97 |

**Abbreviations:** ICD-10, 10^th^ revision of the International Statistical Classification of Diseases and Related Health Problems
