# Supplementary material for: Direct factor Xa inhibitors and the risk of cancer and cancer mortality: A Danish population-based cohort study
Source: PLoS Med. 2024 Jul 1;21(7):e1004400. doi: 10.1371/journal.pmed.1004400 (PMC11251598; doi:10.1371/journal.pmed.1004400)
Supplement: S4 Table — CI, confidence interval; HR, hazard ratio; IPT, inverse probability of treatment; SHR, subdistribution hazard ratio. *Disease stage was recorded in 59.1% of patients with cancer during follow-up. (DOCX) [file pmed.1004400.s005.docx]

**S4 Table.** Subdistributional hazard ratios adjusted for calendar year (intention-to-treat), cause-specific hazard ratio (intention-to-treat analysis), and adjusted hazard ratios (time-varying analysis) for different outcomes in the factor Xa inhibitor versus dabigatran cohorts

|  | **Intention-to-treat analysis** | | **Time-varying analysis** |
| --- | --- | --- | --- |
| **Outcome** | **subdistributional HR adjusted for calendar year (95% CI)** | **Cause-specific HR (95% CI)** | **Adjusted HR (95% CI)** |
| Cancer total | 0.94 (0.88,1.00) | 0.97 (0.89,1.05) | 1.05 (0.95,1.15) |
| Metastatic disease at diagnosis* | 0.96 (0.77,1.21) | 1.07 (0.88,1.30) | 1.02 (0.82,1.27) |
| Cancer-specific mortality | 1.01 (0.91,1.12) | 1.06 (0.94,1.20) | 1.16 (1.00,1.34) |
| All-cause mortality | 1.21 (1.16,1.25) | 1.17 (1.12,1.23) | 1.29 (1.22,1.36) |
| Gastro-intestinal bleeding | 0.79 (0.73,0.86) | 0.83 (0.74,0.92) | 0.86 (0.77,0.97) |
| **Cancer groups** |  |  |  |
| Obesity-related cancer | 1.10 (0.97,1.25) | 1.07 (0.91,1.26) | 1.20 (1.00,1.44) |
| Hormone-related cancer | 1.06 (0.92,1.22) | 1.05 (0.88,1.25) | 1.11 (0.91,1.35) |
| Smoking- and alcohol-related  cancers | 0.74 (0.65,0.85) | 0.86 (0.72,1.01) | 0.84 (0.70,1.02) |
| Immune-related cancer | 1.32 (1.00,1.74) | 1.22 (0.86,1.73) | 1.74 (1.16,2.63) |
| Neurological cancer | 0.98 (0.70,1.36) | 1.05 (0.69,1.61) | 1.12 (0.69,1.82) |
| Other cancers | 0.86 (0.54,1.36) | 1.04 (0.62,1.76) | 0.98 (0.54,1.76) |
| **Cancer types** |  |  |  |
| Colorectal | 1.02 (0.87,1.20) | 0.99 (0.80,1.22) | 1.14 (0.90,1.44) |
| Lung | 0.74 (0.63,0.88) | 0.87 (0.70,1.08) | 0.82 (0.64,1.04) |
| Prostate | 1.10 (0.91,1.33) | 1.04 (0.82,1.32) | 1.07 (0.82,1.39) |
| Breast | 0.95 (0.76,1.19) | 1.04 (0.79,1.38) | 1.11 (0.80,1.53) |
| Hematological | 0.68 (0.55,0.85) | 0.68 (0.52,0.89) | 0.85 (0.63,1.15) |
| Urogenital | 1.02 (0.77,1.36) | 1.12 (0.79,1.58) | 1.25 (0.84,1.85) |
| Gynecological | 1.70 (1.18,2.45) | 1.30 (0.83,2.04) | 1.52 (0.91,2.53) |
| Gastro-esophageal | 0.56 (0.40,0.78) | 0.64 (0.41,1.01) | 0.80 (0.49,1.30) |
| Hepatobiliary | 0.77 (0.44,1.35) | 0.79 (0.40,1.54) | 0.82 (0.38,1.78) |
| Brain | 0.76 (0.34,1.67) | 0.68 (0.29,1.60) | 1.22 (0.46,3.22) |

**Abbreviations:** IPT, inverse probability of treatment; CI, confidence interval; HR, hazard ratio. *****Disease stage was recorded in 59.1% of patients with cancer during follow-up.
