# Supplementary material for: Direct factor Xa inhibitors and the risk of cancer and cancer mortality: A Danish population-based cohort study
Source: PLoS Med. 2024 Jul 1;21(7):e1004400. doi: 10.1371/journal.pmed.1004400 (PMC11251598; doi:10.1371/journal.pmed.1004400)
Supplement: S5 Table — CI, confidence interval; HR, hazard ratio; IPT, inverse probability of treatment; SHR, subdistribution hazard ratio. *Disease stage was recorded in 59.1% of patients with cancer during follow-up. (DOCX) [file pmed.1004400.s006.docx]

**S5 Table.** Subdistributional hazard ratios in the on-treatment analysis for different outcomes in the factor Xa inhibitor versus dabigatran cohorts

| **Outcome** | **Factor Xa inhibitor cohort (n=23711) total (%)** | **Dabigatran cohort (n=23715) total (%)** | **IPT-weighted  subdistributional HR (95% CI)** |
| --- | --- | --- | --- |
| Cancer total | 1694 (7.14) | 1512 (6.37) | 0.99 (0.92,1.06) |
| Metastatic disease at diagnosis* | 336 (1.42) | 279 (1.18) | 1.08 (0.92,1.26) |
| Cancer-specific mortality | 523 (2.21) | 448 (1.89) | 1.01 (0.89,1.14) |
| All-cause mortality | 4693 (19.79) | 3390 (14.3) | 1.24 (1.18,1.29) |
| Gastro-intestinal bleeding | 1021 (4.30) | 1096 (4.62) | 0.83 (0.76,0.90) |
| **Cancer groups** |  |  |  |
| Obesity-related cancer | 526 (2.22) | 405 (1.71) | 1.15 (1.01,1.31) |
| Hormone-related cancer | 405 (1.71) | 337 (1.42) | 1.05 (0.91,1.21) |
| Smoking- and alcohol-related  cancers | 402 (1.70) | 439 (1.85) | 0.81 (0.71,0.93) |
| Immune-related cancer | 112 (0.47) | 58 (0.25) | 1.64 (1.19,2.25) |
| Neurological cancer | 71 (0.30) | 63 (0.27) | 1.00 (0.72,1.41) |
| Other cancers | 46 (0.19) | 37 (0.16) | 1.07 (0.70,1.64) |
| **Cancer types** |  |  |  |
| Colorectal | 301 (1.27) | 255 (1.08) | 1.17 (0.99,1.39) |
| Lung | 256 (1.08) | 262 (1.11) | 0.97 (0.82,1.16) |
| Prostate | 218 (0.92) | 199 (0.84) | 1.09 (0.90,1.32) |
| Breast | 163 (0.69) | 120 (0.51) | 1.36 (1.07,1.72) |
| Hematological | 133 (0.56) | 171 (0.72) | 0.68 (0.54,0.86) |
| Urogenital | 111 (0.47) | 87 (0.37) | 1.27 (0.96,1.69) |
| Gynecological | 76 (0.32) | 47 (0.20) | 1.60 (1.11,2.30) |
| Gastro-esophageal | 53 (0.22) | 60 (0.25) | 0.87 (0.60,1.26) |
| Hepatobiliary | 15 (0.06) | 25 (0.11) | 0.57 (0.30,1.08) |
| Brain | 13 (0.06) | 20 (0.08) | 0.67 (0.33,1.34) |

**Abbreviations:** IPT, inverse probability of treatment; CI, confidence interval; HR, hazard ratio. *****Disease stage was recorded in 59.1% of patients with cancer during follow-up.
