# Supplementary material for: Direct factor Xa inhibitors and the risk of cancer and cancer mortality: A Danish population-based cohort study
Source: PLoS Med. 2024 Jul 1;21(7):e1004400. doi: 10.1371/journal.pmed.1004400 (PMC11251598; doi:10.1371/journal.pmed.1004400)
Supplement: S6 Table — CI, confidence interval; HR, hazard ratio; IPT, inverse probability of treatment; SHR, subdistribution hazard ratio. (DOCX) [file pmed.1004400.s007.docx]

**S6 Table.** Sensitivity analysis with IPT-weighted cumulative incidence and subdistribution hazard ratios for different outcomes in the factor Xa inhibitor cohort versus the dabigatran cohort during 9 years of follow-up.

|  | **Inclusion period 2011 – 2015 9 years of follow-up** | | | |
| --- | --- | --- | --- | --- |
|  | **Factor Xa inhibitors (n=23711) total (%)** | **Dabigatran (n=23715) total (%)** | **IPT-weighted Subdistributional HR** | **P-value** |
| Cancer total | 2277 (9.60) | 2706 (11.41) | 0.88 (0.83,0.93) | <.0001 |
| Metastatic disease at diagnosis | 448 (1.89) | 480 (2.03) | 0.97 (0.85,1.10) | 0.5932 |
| Cancer-specific mortality | 1166 (4.92) | 1378 (5.81) | 0.94 (0.87,1.01) | 0.1031 |
| All-cause mortality | 8357 (35.25) | 8895 (37.51) | 1.13 (1.09,1.16) | <.0001 |
| Gastro-intestinal bleeding | 1429 (6.03) | 1916 (8.08) | 0.80 (0.75,0.86) | <.0001 |
| **Cancer groups** |  |  |  |  |
| Obesity-related cancer | 675 (2.85) | 722 (3.04) | 0.98 (0.88,1.08) | 0.6378 |
| Hormone-related cancer | 573 (2.42) | 640 (2.70) | 0.95 (0.85,1.07) | 0.3933 |
| Smoking- and alcohol-related  cancers | 539 (2.27) | 696 (2.93) | 0.80 (0.71,0.89) | <.0001 |
| Immune-related cancer | 141 (0.60) | 156 (0.66) | 0.97 (0.77,1.21) | 0.7634 |
| Neurological cancer | 89 (0.38) | 102 (0.43) | 0.92 (0.70,1.22) | 0.5663 |
| Other cancers | 64 (0.27) | 62 (0.26) | 1.05 (0.74,1.49) | 0.7985 |
| **Cancer types** |  |  |  |  |
| Colorectal | 387 (1.63) | 440 (1.86) | 0.92 (0.81,1.06) | 0.2525 |
| Lung | 316 (1.33) | 399 (1.68) | 0.81 (0.70,0.94) | 0.0056 |
| Prostate | 321 (1.35) | 361 (1.52) | 0.94 (0.81,1.09) | 0.4093 |
| Breast | 222 (0.94) | 256 (1.08) | 0.94 (0.79,1.13) | 0.5152 |
| Hematological | 196 (0.83) | 327 (1.38) | 0.63 (0.53,0.75) | <.0001 |
| Urogenital | 155 (0.65) | 158 (0.67) | 1.03 (0.83,1.28) | 0.7934 |
| Gynecological | 88 (0.37) | 84 (0.35) | 1.09 (0.81,1.46) | 0.5797 |
| Gastro-esophageal | 66 (0.28) | 110 (0.47) | 0.61 (0.45,0.83) | 0.0014 |
| Hepatobiliary | 29 (0.12) | 46 (0.19) | 0.66 (0.41,1.04) | 0.0717 |
| Brain | 19 (0.08) | 35 (0.15) | 0.60 (0.35,1.04) | 0.0701 |

**Abbreviations:** IPT, inverse probability of treatment; CI, confidence interval; HR, hazard ratio.
