# Supplementary material for: Direct factor Xa inhibitors and the risk of cancer and cancer mortality: A Danish population-based cohort study
Source: PLoS Med. 2024 Jul 1;21(7):e1004400. doi: 10.1371/journal.pmed.1004400 (PMC11251598; doi:10.1371/journal.pmed.1004400)
Supplement: S7 Table — CI, confidence interval; HR, hazard ratio; IPT, inverse probability of treatment; SHR, subdistribution hazard ratio. (DOCX) [file pmed.1004400.s008.docx]

**S7 Table.** Sensitivity analysis with IPT-weighted cumulative incidence and subdistribution hazard ratios for different outcomes in the factor Xa inhibitor cohort versus the dabigatran cohort during 5 years of follow-up with inclusion period between 2011 and 2014.

|  | **Inclusion period 2011 – 2014 5 years of follow-up** | | | |
| --- | --- | --- | --- | --- |
|  | **Factor Xa inhibitors (n=15772) total (%)** | **Dabigatran (n=15801) total (%)** | **IPT-weighted Subdistributional HR** | **P-value** |
| Cancer total | 1547 (9.81) | 1516 (9.59) | 1.02 (0.95,1.10) | 0.5152 |
| Metastatic disease at diagnosis | 302 (1.91) | 271 (1.72) | 1.12 (0.95,1.32) | 0.1886 |
| Cancer-specific mortality | 737 (4.67) | 642 (4.06) | 1.15 (1.04,1.28) | 0.0079 |
| All-cause mortality | 4670 (31.51) | 4229 (26.76) | 1.22 (1.17,1.27) | <.0001 |
| Gastro-intestinal bleeding | 907 (5.75) | 980 (6.20) | 0.93 (0.85,1.01) | 0.0999 |
| **Cancer groups** |  |  |  |  |
| Obesity-related cancer | 465 (2.95) | 415 (2.62) | 1.12 (0.98,1.28) | 0.0831 |
| Hormone-related cancer | 383 (2.43) | 345 (2.18) | 1.11 (0.96,1.29) | 0.1522 |
| Smoking- and alcohol-related  cancers | 349 (2.21) | 401 (2.54) | 0.87 (0.76,1.01) | 0.0629 |
| Immune-related cancer | 110 (0.70) | 73 (0.46) | 1.51 (1.12,2.03) | 0.0066 |
| Neurological cancer | 50 (0.32) | 59 (0.37) | 0.84 (0.58,1.23) | 0.3768 |
| Other cancers | 51 (0.33) | 36 (0.23) | 1.44 (0.94,2.21) | 0.0942 |
| **Cancer types** |  |  |  |  |
| Colorectal | 254 (1.61) | 254 (1.61) | 1.00 (0.84,1.19) | 0.9731 |
| Lung | 210 (1.33) | 228 (1.44) | 0.92 (0.76,1.11) | 0.3938 |
| Prostate | 223 (1.41) | 203 (1.29) | 1.10 (0.91,1.33) | 0.3421 |
| Breast | 139 (0.88) | 127 (0.80) | 1.10 (0.87,1.40) | 0.4353 |
| Hematological | 140 (0.89) | 187 (1.18) | 0.75 (0.60,0.93) | 0.0097 |
| Urogenital | 109 (0.69) | 83 (0.53) | 1.31 (0.98,1.74) | 0.0642 |
| Gynecological | 66 (0.42) | 48 (0.30) | 1.37 (0.95,1.99) | 0.0930 |
| Gastro-esophageal | 31 (0.20) | 67 (0.43) | 0.46 (0.30,0.71) | 0.0004 |
| Hepatobiliary | 13 (0.08) | 27 (0.17) | 0.48 (0.25,0.93) | 0.0296 |
| Brain | 14 (0.09) | 18 (0.11) | 0.77 (0.38,1.56) | 0.4664 |

**Abbreviations:** IPT, inverse probability of treatment; CI, confidence interval; HR, hazard ratio.
