# Supplementary material for: Direct factor Xa inhibitors and the risk of cancer and cancer mortality: A Danish population-based cohort study
Source: PLoS Med. 2024 Jul 1;21(7):e1004400. doi: 10.1371/journal.pmed.1004400 (PMC11251598; doi:10.1371/journal.pmed.1004400)
Supplement: S1 Fig — CHF, congestive heart failure; COPD, chronic obstructive pulmonary disease; IBD, inflammatory bowel disease; Istroke, ischemic stroke; MI, myocardial infarction; NSAIDs, nonsteroidal anti-inflammatory drugs; prop score, propensity score; obs, observations; w3m, within 3 months. (DOCX) [file pmed.1004400.s009.docx]

**S1 Figure.** Standardized differences for covariates included in the propensity score model


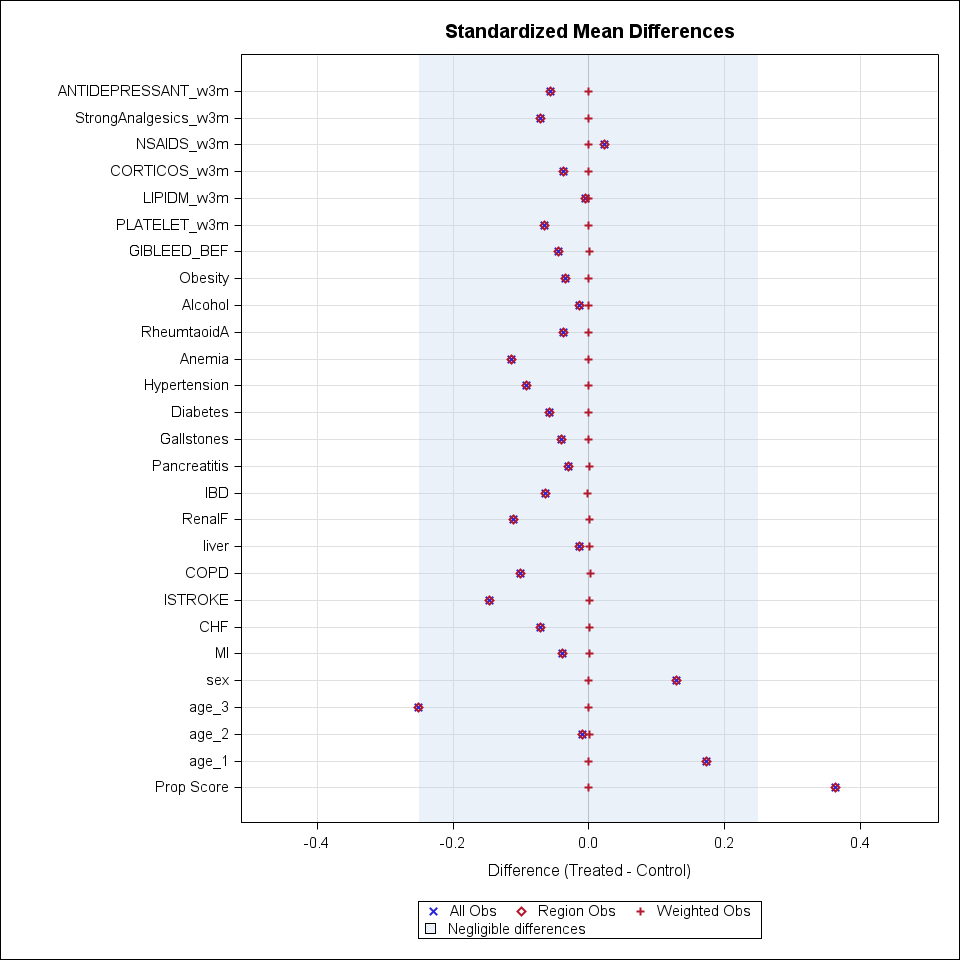


**Abbreviations:** w3m, within 3 months; NSAIDs, non-steroidal anti-inflammatory drugs; IBD, inflammatory bowel disease; COPD, chronic obstructive pulmonary disease; Istroke, ischemic stroke; CHF, congestive heart failure; MI, myocardial infarction; prop score, propensity score; obs, observations.
