# Supplementary material for: Direct factor Xa inhibitors and the risk of cancer and cancer mortality: A Danish population-based cohort study
Source: PLoS Med. 2024 Jul 1;21(7):e1004400. doi: 10.1371/journal.pmed.1004400 (PMC11251598; doi:10.1371/journal.pmed.1004400)
Supplement: S2 Fig — Bars represent histogram of propensity scores in the 2 cohorts (expressed as percentages per 100) and lines represent kernel density plots of propensity scores prior to inverse probability of treatment weighting. (DOCX) [file pmed.1004400.s010.docx]

**S2 Figure.** Propensity score distribution in the factor Xa inhibitor and dabigatran cohorts


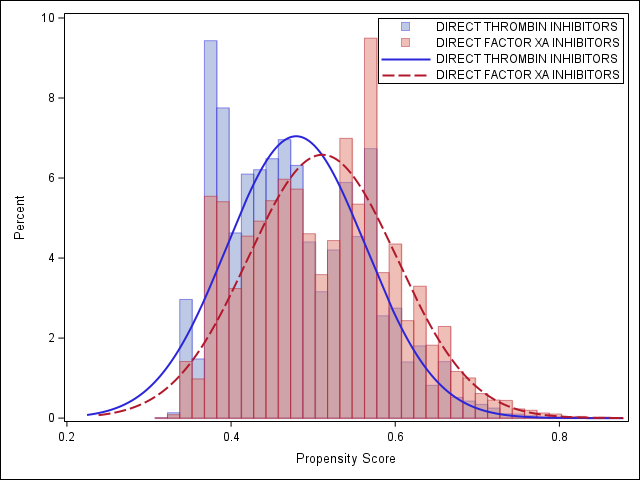


Bars represent histogram of propensity scores in the two cohorts (expressed as percentages per 100) and lines represent kernel density plots of propensity scores prior to inverse probability of treatment weighting.
