# Supplementary material for: Direct factor Xa inhibitors and the risk of cancer and cancer mortality: A Danish population-based cohort study
Source: PLoS Med. 2024 Jul 1;21(7):e1004400. doi: 10.1371/journal.pmed.1004400 (PMC11251598; doi:10.1371/journal.pmed.1004400)
Supplement: S1 Appendix — (DOCX) [file pmed.1004400.s011.docx]

**Direct factor Xa inhibitors and the risk of cancer onset and mortality: proposal for a nationwide cohort study**

**July 2021**

**Investigators**

Floris Bosch, Erzsébet Horváth-Puhó, Nick van Es, Suzanne Cannegieter, Henrik Toft Sørensen.

# INTRODUCTION AND RATIONALE

Cancer is associated with an activation of the hemostatic system, which, in turn, has been suggested to promote tumor growth and metastasis.^1^ An inhibitory effect of oral anticoagulants on cancer progression and survival in humans was first suggested in 1992 by Prandoni et al,^2^ who found a reduced overall mortality among patients with cancer and with venous thromboembolism (VTE), initially treated with LMWH relative to unfractionated heparin. However, in a randomized trial on the effect of LMWH to the survival of patients with cancer, no overall survival benefit for those treated with LMWH was observed.^3^ After this negative result was published in 2011, the interest of LMWH to decrease cancer-related morbidity and mortality diminished. Recently renewed interest in the hypothesis that oral anticoagulants can decrease cancer risk was sparked by results from Graf et al. They found (in a mouse model) that the efficacy of the oral anticoagulant rivaroxaban (a direct factor Xa inhibitor) is comparable with anti-programmed cell death ligand 1 (PD-L1) therapy and that rivaroxaban synergizes with anti-PD-L1 in improving antitumor immunity.^4^ This finding suggests that rivaroxaban has anti-cancer opportunities in humans by enhancing infiltration of dendritic cells and cytotoxic T cell at the tumor site, which promotes antitumor immunity. In the current study proposal, our aim is to determine whether use of a direct factor Xa inhibitor (including rivaroxaban) is associated with decreased cancer onset and/or cancer mortality as compared with a direct thrombin inhibitor in a nationwide observational study.

# OBJECTIVES

To evaluate whether patients who use direct factor Xa inhibitors for prevention of ischemic stroke in atrial fibrillation are at lower risk of developing cancer and/or cancer mortality compared with those who are treated with the direct thrombin inhibitor dabigatran.

# METHODS

We propose a clinical trial setting in an observational new user, active comparator design. In such a study design, cohorts of new drug users (ie, patients newly prescribed an index drug A and patients newly prescribed a therapeutic alternative or comparator drug B are assembled and followed over time for the health outcome(s) of interest.^5^ Confounding (by indication) will be minimal under the assumption that there is no preference for a type of direct oral anticoagulant (DOAC; either direct factor Xa inhibitor or direct thrombin inhibitor) to be prescribed for any particular patient. This assumption seems valid as, according to clinical guidelines, all DOACs are equally effective and safe to be used in patients with atrial fibrillation.^6,7^

Therefore, the purpose of this observational study is to quantify the association between DOAC use (direct factor Xa inhibitors versus direct thrombin inhibitors) and new onset of cancer/ cancer mortality in a general population by using an active comparator, new user design. Since the effect of rivaroxaban to cancer onset in the mouse model from Graf et al is assumed to be anti-FXa specific,^4^ we hypothesize that cancer onset and death from cancer is less common in direct factor Xa inhibitors users than in direct thrombin inhibitors users.

Follow-up starts at the admission date of the inpatient or outpatient AF diagnosis. DOAC drug use will be defined in 3 ways: A) in an intention to treat fashion, i.e. from the admission date of the AF diagnosis until end of study. B) In an on-treatment fashion, i.e. from the admission date of the AF diagnosis until the date that the initial DOAC has not been prescribed for >200 days or until the end of study, whichever occurs first. C) in an time-dependant fashion.

# STUDY POPULATION

The study aims to include all patients in Denmark ≥18 years of age with a first-time primary or secondary hospital inpatient or outpatient discharge diagnosis of atrial fibrillation or flutter registered in the DNPR between September 2011 until October 2019. September 2011 is chosen since by that time to results of the RE-LY trial (dabigatran)^9^ and the ROCKET-AF (rivaroxaban)^10^ trial were published and both drugs were approved for the use of stroke prevention in patients with atrial fibrillation by the European Medicines Agency.^6^ We specifically choose to only include atrial fibrillation patients as they are candidates for prolonged (usually lifelong) use of anticoagulation. All patients in whom DOACs were started in the 3 months prior or after the first AF diagnosis can be included. Outpatients with a secondary diagnosis of AF and more than one year between admission and discharge will be excluded.

Patients with an active cancer diagnosis other than non-melanoma skin cancer at the time of first onset of atrial fibrillation will be excluded. Patients with previous anticoagulation prescriptions for other diagnoses (e.g. venous thromboembolism) can be included.

# REGISTRIES

Data on diagnoses, comorbidities and medical history will be collected from the Danish National Patient Registries (DNPR). The DNPR collects data on all hospitalizations in Denmark since 1977. Data on outpatient and emergency department visits are recorded since 1995. From January 1994 and onwards diagnoses are coded via the 10*^th^ revision of the International Statistical Classification of Diseases and Related Health Problems* (ICD-10).^11^ Data on prescribed medication will be collected from the Danish Health Service Prescription Database (DNHSPD) using the Anatomical Therapeutic Chemical (ATC) coding system.^12^ This database records the ATC codes, date of prescription and defined daily dosage. Date and cause of death will be collected from the Danish Register of Causes of Death, which records all causes of death in Denmark since 1875. Since 1994 all causes of death are recorded by ICD-10 codes.^13^ Date of death will be recorded from the Civil Registration System. Cancer diagnosis is recorded from the Danish Cancer Registry. This registry records all diagnoses of cancer in the Danish population. Diagnoses are recorded with the ICD-10 coding system from 1978 and onwards.^14^ The data from the different databases will be linked through the unique Civil Registration Number, given to every resident in Denmark upon birth or immigration in the Civil Registration System. The Civil Registration System holds information on date of birth, gender and date of death or emigration, for all Danish residents.^15^

## Data collection

### Atrial fibrillation

All patient with a first-time in- or outpatient diagnosis of AF in the DNPR will be recorded according to the ICD-10 coding system. The positive predictive value of AF in the DNPR has been described previously and showed value of 92.6%^16^.

### Anticoagulation treatment

Data on dose and date of prescription of different DOACs will be collected. The types of DOAC and their ATC codes collected from the DNHSPD is shown in Table 2.

### Cancer diagnosis and cancer mortality

Cancer diagnosis will be collected from the Danish Cancer Registry via ICD-10 codes. All malignant cancers will be recorded except for non-melanoma skin cancer (C44 and C4A).

The ICD-10 codes used for the cancer diagnosis are presented in table 1. Cancer mortality will be collected from the Danish Register of Causes of Death by ICD-10 codes.

### Comorbidities and co-medication

The following comorbidities and co-medication at baseline will be collected to adjust for confounders: age, sex, chronic liver disease, chronic kidney disease, previous TIA or stroke, use of platelet aggregation inhibitors, CHA₂DS₂-VASc score. Comorbidities will be collected from the DNPR via ICD-10 codes (Table 1) and co-medication will be collected from the DNHSPD (Table 2). The CHA₂DS₂-VASc score will be calculated with the corresponding ICD-10 codes. Patients with one or more anti-diabetes drugs or two or more antihypertensive drugs at baseline will be recorded as having a history of diabetes or hypertension respectively, regardless of the presence of a corresponding ICD-10 code.

# OUTCOMES

## Main study outcome

- First diagnosis of cancer other than non-melanoma skin cancer

## Secondary study outcome

- Cancer group diagnosis
- Cancer mortality
- All-cause mortality

# STATISTICAL ANALYSIS

Propensity scores will be computed with a multivariable logistic regression model with DOAC type as the dependent variable (factor Xa inhibitors vs. dabigatran), including the following covariates: age; sex; myocardial infarction; congestive heart failure; ischemic stroke; chronic obstructive pulmonary disease; liver disease; renal disease; inflammatory bowel disease; pancreatitis; gallstones; diabetes mellitus (including use of diabetes medication); hypertension; anemia; rheumatoid arthritis; alcohol dependency (including drugs for alcohol dependency); obesity and obesity-related disorders; and use of platelet aggregation inhibitors, antihypertensive agents, lipid lowering drugs, glucocorticoids, NSAIDs, strong analgesics, and antidepressants.

Using the propensity score weighted cohorts, cumulative incidence curves will be constructed for the outcomes of cancer, all-cause mortality, and gastrointestinal bleeding. For the primary and secondary outcomes, inverse probability of treatment weighted (IPTW) Fine and Gray competing risk regression models will be used to calculate subdistribution hazard ratios (SHRs), with overall death as a competing event.

Three sensitivity analyses will be performed to assess outcomes in:

- An intention-to-treat analysis, adjusted for calendar year
- An on-treatment analysis
- A time-varying exposure approach.

# SAMPLE SIZE ESTIMATES

In Europe, approximately 2% of individuals have atrial fibrillation, of whom the majority uses an oral anticoagulant [10]. It is estimated (in the Danish Diet, Cancer and Health study) that approximately 9% of patients with (new onset) atrial fibrillation will develop cancer within 4 years [10]. With a population of n=5.6 million, we expect to include 5.6*0.02=112 000 patients with atrial fibrillation each year (from 2011-2019) with a mean follow-up of 4 years, which corresponds to 448 000 person years. We expect that of those, 50% use a DOAC or 224 000 person years over the total follow-up. Of those 224 000 person years, a third is expected to use direct thrombin inhibitor (75 000 person years) and the others direct factor Xa inhibitors (150 000 person years or n=37500 followed for 4 years). WITH n=37500 in the exposed group, a risk of cancer in the unexposed group of 9% and an unexposed/ exposed ratio of 1/2, we should be able to find a 8% reduction of cancer (relative risk 0.92), with a 2-sided alpha and >80% power.


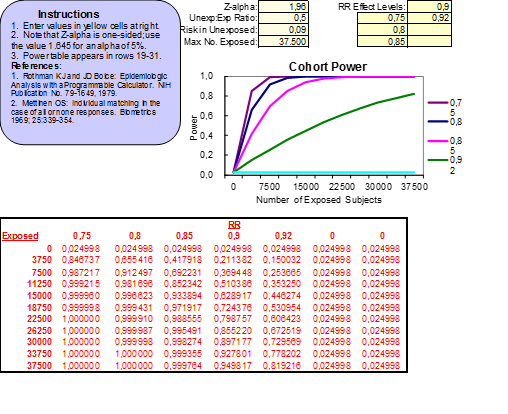


# TIME TABLE

December 2020: Approval of the protocol including selection criteria, ICD codes, empty
 tables

February 2021: Perform the requested analyses

March-May 2021: Write a first draft of the outcomes and present results in a meeting

# REFERENCES

1. Rondon AMR, Kroone C, Kapteijn MY, Versteeg HH, Buijs JT. Role of Tissue Factor in Tumor Progression and Cancer-Associated Thrombosis. *Semin Thromb Hemost*. 2019;45(04):396-412.

2. Prandoni P, Carta M, Cogo A, et al. Comparison of subcutaneous low-molecular-weight heparin with intravenous standard heparin in proximal deep-vein thrombosis. *Lancet*. 1992;339(8791):441-445.

3. van Doormaal FF, Di Nisio M, Otten H-M, Richel DJ, Prins M, Buller HR. Randomized Trial of the Effect of the Low Molecular Weight Heparin Nadroparin on Survival in Patients With Cancer. *J Clin Oncol*. 2011;29(15):2071-2076.

4. Graf C, Wilgenbus P, Pagel S, et al. Myeloid cell–synthesized coagulation factor X dampens antitumor immunity. *Sci Immunol*. 2019;4(39):eaaw8405.

5. Lund JL, Richardson DB, Stürmer T. The Active Comparator, New User Study Design in Pharmacoepidemiology: Historical Foundations and Contemporary Application. *Curr Epidemiol Reports*. 2015;2(4):221-228.

6. Camm AJ, Lip GYH, De Caterina R, et al. 2012 focused update of the ESC Guidelines for the management of atrial fibrillation. *Eur Heart J*. 2012;33(21):2719-2747.

7. Kirchhof P, Benussi S, Kotecha D, et al. 2016 ESC Guidelines for the management of atrial fibrillation developed in collaboration with EACTS. *Eur Heart J*. 2016;37(38):2893-2962.

8. Vinter N, Christesen AMS, Fenger‐Grøn M, Tjønneland A, Frost L. Atrial Fibrillation and Risk of Cancer: A Danish Population‐Based Cohort Study. *J Am Heart Assoc*. 2018;7(17).

9. Connolly SJ, Ezekowitz MD, Yusuf S, et al. Dabigatran versus Warfarin in Patients with Atrial Fibrillation. *N Engl J Med*. 2009;361(12):1139-1151.

10. Patel MR, Mahaffey KW, Garg J, et al. Rivaroxaban versus Warfarin in Nonvalvular Atrial Fibrillation. *N Engl J Med*. 2011;365(10):883-891.

11. Schmidt M, Schmidt SAJ, Sandegaard JL, Ehrenstein V, Pedersen L, Sørensen HT. The Danish National patient registry: A review of content, data quality, and research potential. *Clin Epidemiol*. 2015;7:449-490.

12. Pottegård A, Schmidt SAJ, Wallach-Kildemoes H, Sørensen HT, Hallas J, Schmidt M. Data resource profile: The Danish national prescription registry. *Int J Epidemiol*. 2017;46(3):798.

13. Helweg-Larsen K. The Danish Register of Causes of Death. *Scand J Public Health*. 2011;39(7_suppl):26-29.

14. Gjerstorff ML. The Danish Cancer Registry. *Scand J Public Health*. 2011;39(7_suppl):42-45.

15. Schmidt M, Pedersen L, Sørensen HT. The Danish Civil Registration System as a tool in epidemiology. *Eur J Epidemiol*. 2014;29(8):541-549.

16. Rix TA, Riahi S, Overvad K, Lundbye-Christensen S, Schmidt EB, Joensen AM. Validity of the diagnoses atrial fibrillation and atrial flutter in a Danish patient registry. *Scand Cardiovasc J*. 2012;46(3):149-153.

17. Søgaard M, Heide-Jørgensen U, Nørgaard M, Johnsen SP, Thomsen RW. Evidence for the low recording of weight status and lifestyle risk factors in the Danish National Registry of Patients, 1999–2012. *BMC Public Health*. 2015;15(1):1320.

**Table 1. cancer types and groups**

| **Cancers** | **ICD-10 codes** |
| --- | --- |
| *All Cancer* | C00-C96 (excluding C44 and C4A) |
|  |  |
| *Obesity-related cancers* |  |
| Esophagus | C15 |
| Pancreas | C25 |
| Colon including the rectosigmoid junction | C18-C19 |
| Rectum | C20 |
| Breast, postmenopausal (≥60 years) | C50 |
| Uterus | C54-C55 |
| Kidney | C64 |
| Gallbladder and bile ducts | C23-C24 |
| Thyroid gland | C73 |
| *Smoking- and alcohol-related cancers* |  |
| Lip | C00 |
| Tongue | C01-02 |
| Mouth | C03-06 |
| Tonsil and pharynx | C09-C13 |
| Other and poorly specified locations in lip, oral cavity, and pharynx | C14 |
| Larynx | C32 |
| Other and poorly specified locations in airways and respiratory organs | C39 |
| Stomach | C16 |
| Small intestine | C17 |
| Liver, including intrahepatic bile ducts | C22 |
| Lung, bronchus and trachea | C33-C34 |
| Renal pelvis | C65 |
| Ureter | C66 |
| Urinary bladder | C67 |
| *Hematological cancers* |  |
| Hodgkin’s lymphoma (included morphologic code 965-966) | C81 |
| Non-Hodgkin’s lymphoma, excluding leukemia and myelomatosis (including morphologic code 959, 967-972) | C82-85 |
| Malignant myeloproliferative disease | C88 |
| Multiple myeloma and other plasma cell neoplasms | C90 |
| Myeloid leukemia | C92 |
| Lymphocytic leukemia | C91 |
| Monocytic leukemia | C93 |
| Other leukemia | C94-95 |
| Other and unspecified cancers of lymphoid, hematopoietic, and related tissues | C96 |
| Metastasis and unspecified cancer in lymph nodes (only when no primary tumor is coded) | C77-79 (only if no primary tumor is coded) |
| *Immune-related cancers* |  |
| Anus and anal canal, excluding malignant melanomas (morphologic code 872-879) and basal cell cancers (morphologic code 809) | C21 |
| Cervix | C53 |
| External female genitalia, excluding basal cell carcinomas (morphological code 809) | C51 |
| Penis, excluding basal cell carcinomas (morphological code 809) | C60 |
| Malignant melanoma, including those located in anus and anal canal (morphological code 872-879) | C43 |
| Non-melanoma skin cancers, excluding basal cell carcinoma (morphologic code 809) | C44 |
| *Cancers of neurological origin* |  |
| Meningioma | C70, D32, D42 |
| Brain, including hypophysis, corpus pineale, and ductus craniopharyngealis | C71, C751-753, D330-D332, D352-D354, D430-D432, D443-D445 |
| Spinal cord, cranial nerves, and other parts of central nervous system | C72, D333-D339, D433-D439 |
| *Hormone-related cancers* |  |
| Prostate | C61 |
| Testicular | C62 |
| Vagina, excluding basal cell carcinomas (morphological code 809) | C52 |
| Breast, premenopausal (<60 years) | C50 |
| Ovary and fallopian tube | C56, C570-574 |
| *All other cancers* |  |
| Salivary glands | C07-08 |
| Other and ill-defined cancers of digestive organs | C26 |
| Nasal cavity, middle ear, and accessory sinuses | C30-C31 |
| Thymus | C37 |
| Heart and mediastinum | C381-383, C388 |
| Pleura, including mesothelioma pleura | C384, C450 |
| Bone and articular cartilage | C40-C41 |
| Kaposi’s sarcoma | C46, B210 |
| Mesothelioma | C45·1-C45·9 |
| Peripheral nerves and autonomic nervous system | C47 |
| Retroperitoneum and peritoneum | C48 |
| Malignant neoplasm of other connective and soft tissue | C49 |
| Placenta | C58 |
| Other and unspecified cancers in female genital organs | C577-579 |
| Other and unspecified cancers in male genital organs, excluding basal cell carcinomas (morphological code 809) | C63 |
| Other and unspecified cancers in urinary organs | C68 |
| Eye and adnexa |  |
| Adrenal gland | C74 |
| Other endocrine structures, excluding hypophysis, corpus pineale, and ductus craniopharyngealis | C750, C754-759 |
| Malignant neoplasm at other, ill-defined, or unspecified sites | C76, C80 |
| Malignant neoplasms at independent (primary) multiple sites | C97 |

**Table 2. covariates**

| **Diagnosis** | **ICD-8 code** | **ICD-10 code** |
| --- | --- | --- |
| Myocardial infarction | 410 | I21;I22;I23 |
| Congestive heart failure | 42709, 42710, 42711, 42719, 42899, 78249 | I500, I501, I502, I503, I508, I509, I110, I130, I132, I420, I426, I427, I428, I429 |
| Ischemic stroke | 433-434 | I63 |
| Chronic obstructive pulmonary  disease | 491; 492 | J40-J44 |
| Liver disease | 070.00; 070.02; 070.04; 070.06; 070.08; 456.00-456.09; 571; 573.00; 573.01; 573.04 | B15.0; B16.0; B16.2; B18; B19.0; K70.0-K70.3; K70.4; K70.9; K71-K74; K76.0; K76.6; I85 |
| Renal disease | 403; 404; 580-583; 584; 590.09; 593.19; 753.10-753.19; 792 | I12; I13; N00-N05; N07; N11; N14; N17-N19; Q61 |
| Inflammatory bowel disease | 563 | K50-K52 |
| Pancreatitis | 577.0; 577.1; 577.10 | K85; K86.0; K86.1 |
| Gallstones | 574 | K80 |
| Diabetes mellitus | 249.00, 249.06, 249.07, 249.09, 250.00, 250.06, 250.07, 250.09 | E10-E13;  O24 (except O24.4), G63.2, H36.0, N08.3 |
| Rheumatoid Arthritis | 712 | M05; M06 |
| Alcohol dependency | 291; 303; 979; 577.10; 571.09; 571.10 | F10; E24.4; G31.2; G62.1; G72.1; I42.6; K29.2; K70; K86.0; O35.4; Y57.3; Z50.2; Z71.4; Z72.1 |
| Obestiy and obesity related disorders | 277 | E65-E68 |

**Table 3. Drugs and ATC-codes**

| **Drug** | **ATC-code** |
| --- | --- |
| *Direct thrombin inhibitors* |  |
| Dabigatran | B01AE07 |
| *Direct factor-Xa inhibitors* |  |
| Rivaroxaban | B01AF01 |
| Apixaban | B01AF02 |
| Edoxaban | B01AF03 |
| *Drugs as covariates* |  |
| Platelet aggregation inhibitors | B01AC |
| Diabetes medication* | A10 |
| Drugs for alcohol dependency* | N07BB01-3 |
| *PD-L1 inhibitors* |  |
| Durvalumab | L01XC28 |
| Avelumab | L01XC31 |
| Atezolizumab | L01XC32 |

*Add to diagnosis definition
