# Supplementary material for: Direct factor Xa inhibitors and the risk of cancer and cancer mortality: A Danish population-based cohort study
Source: PLoS Med. 2024 Jul 1;21(7):e1004400. doi: 10.1371/journal.pmed.1004400 (PMC11251598; doi:10.1371/journal.pmed.1004400)
Supplement: Attachment — Submitted filename: Response to the Reviewers - PMEDICINE-D-23-03433R1.docx [file pmed.1004400.s012.docx]

ACADEMIC EDITOR COMMENTS

My review of this paper aligns most closely with Reviewer 1. This is a well-designed, well-executed negative study. The sentence in the abstract, discussion, and cover letter "subgroup analyses suggested that it might decrease the risk of several cancer types" is somewhat exaggerated -- there were three cancer types (out of 10) where cancer incidence was lower in the Factor Xa inhibitor group (Table 2), but these data did not consistently hold up in the Table S4 analysis.

We thank the Editor for the kind words. We agree that the wording of conclusions on subgroup analyses is formulated somewhat strong. In the abstract we changed the conclusions so that it corresponds with the aims in the abstract of assessing cancer incidence and cancer-related mortality. We have removed the subgroup analyses from the results section of the abstract and changed the conclusions of the abstract to:

*“In this population based cohort study, factor Xa inhibitor use was not associated with an overall lower incidence of cancer or cancer-related mortality when compared to dabigatran.”*

We changed the wording in the discussion accordingly and added a more robust discussion on subgroup analysis on page 12.

EDITORIAL COMMENTS

The editors concur with the reviewers that the follow-up is rather short for the outcome of interest. Therefore, we suggest extending the follow-up period to provide a more robust assessment and agree with the statistical reviewer that a sensitivity analysis adjusting for age is merited. In addition, we ask that you report absolute numbers throughout the entire manuscript and revise your discussion to include a more formal discussion of limitations.

We thank the Editor for the comments. According to the study hypothesis, FXa inhibitors should prevent progression of cancer and cancer growth with a mechanism similar to PD-L1 inhibition, which suggests it could also have an effect when a tumor has not yet been diagnosed but already has had several years since inception, possibly resulting in postponement of a tumor becoming clinically apparent. We therefore hypothesized that 5 years would be sufficient to observe a potential effect.

However, we acknowledge that a longer follow-up time could provide important additional information. Therefore, we have now added an analysis in which follow-up time was extended to 9 years total (see S5 Table). We would like to note that this analysis needs to be interpreted cautiously, since the majority of patients in the first period of the study used dabigatran. Therefore, in this analysis up to 9 years, the mean follow-up time is longer in the dabigatran group (5.98 vs 4.60 years), which may introduce bias since patients on dabigatran had more time to develop cancer. This bias is not present in the primary analysis in which all patients were followed for five years (until end of 2020), regardless of time of inclusion. Due to this potential bias, we hope the Editor agrees to add the analysis with 9 years of follow-up only as a sensitivity analysis.

After further discussion in our group, we concluded that we could also not rule out an impact of COVID-19 during the first year of the pandemic, which overlaps with our follow-up. In 2020, there were less cancer diagnosis in Denmark due to the pandemic, possibly resulting in skewed results since in most patients who were still in follow-up in that year were using a factor Xa inhibitor. Also, all-cause mortality was likely impacted by COVID-19. We therefore would like to add another sensitivity analysis in which inclusion stops at the end of 2014 and follow-up ends at the end of 2019 (prior to the COVID-19 pandemic). We have added this analysis to the manuscript and results to the supplements (see S5 Table).

We agree with the Editor and Statistical Reviewer that age is a very important determinant for the development of cancer. The covariate balance plot shows the balance of included variables in the propensity score adjusted model prior to weighting (see S1 Figure). After IPT weighting, there is good balance in the different age groups as shown in Table 1. We added a Table to this response (see below) comparing SHRs adjusted for calendar-year to SHR adjusted for calendar-year and age. Due to the good balance in the pseudo-population after weighting (as used in all analysis in the manuscript), we see no difference between these two groups. We therefore would like to leave this out of the manuscript.

Finally we have reported absolute numbers throughout the entire manuscript as suggested and have now included a formal discussion of limitations in the Discussion section.

| **IPTW-weighted SD-HR (adjusted for calendar year)** | **Pr > ChiSq** | **IPTW-weighted SD-HR (adjusted for calendar year and age)** | **Pr > ChiSq** |
| --- | --- | --- | --- |
| 0.94 (0.88–1.00) | 0.0579 | 0.93 (0.87–1.00) | 0.0423 |
| 1.10 (0.97–1.25) | 0.1202 | 1.10 (0.97–1.24) | 0.1465 |
| 0.74 (0.65–0.85) | <.0001 | 0.74 (0.65–0.85) | <.0001 |
| 0.68 (0.55–0.85) | 0.0005 | 0.68 (0.55–0.84) | 0.0004 |
| 1.32 (1.00–1.74) | 0.0531 | 1.31 (0.99–1.73) | 0.0578 |
| 0.98 (0.70–1.36) | 0.8919 | 0.97 (0.69–1.35) | 0.8549 |
| 1.06 (0.92–1.22) | 0.4139 | 1.06 (0.92–1.22) | 0.4329 |
| 0.86 (0.54–1.36) | 0.5175 | 0.85 (0.54–1.35) | 0.5017 |
| 0.56 (0.40–0.78) | 0.0007 | 0.56 (0.40–0.79) | 0.0007 |
| 1.02 (0.87–1.20) | 0.8193 | 1.00 (0.85–1.18) | 0.9774 |
| 0.77 (0.44–1.35) | 0.3559 | 0.75 (0.43–1.30) | 0.3007 |
| 0.74 (0.63–0.88) | 0.0006 | 0.74 (0.63–0.88) | 0.0005 |
| 0.95 (0.76–1.19) | 0.6716 | 0.93 (0.74–1.17) | 0.5440 |
| 1.70 (1.18–2.45) | 0.0047 | 1.70 (1.18–2.46) | 0.0047 |
| 1.10 (0.91–1.33) | 0.3118 | 1.11 (0.92–1.34) | 0.2756 |
| 1.02 (0.77–1.36) | 0.8768 | 1.02 (0.77–1.36) | 0.8875 |
| 0.76 (0.34–1.67) | 0.4887 | 0.74 (0.34–1.61) | 0.4449 |
| 0.68 (0.55–0.85) | 0.0005 | 0.68 (0.55–0.84) | 0.0004 |
| 0.79 (0.73–0.86) | <.0001 | 0.77 (0.71–0.84) | <.0001 |
| 1.21 (1.16–1.25) | <.0001 | 1.14 (1.09–1.18) | <.0001 |
| 1.01 (0.91–1.12) | 0.8909 | 0.98 (0.88–1.09) | 0.6947 |

GENERAL COMMENTS

All suggestions in General comments, financial disclosure and competing interest have been addressed or added to the manuscript.

1) Please include page numbers and line numbers in the manuscript file. Use continuous line numbers (do not restart the numbering on each page). For review purposes, we started counting the Abstract as page 1.

Page and line numbers have been added to the manuscript.

2) Please cite the reference numbers in square brackets (e.g., "We used the techniques developed by our colleagues [19] to analyze the data"). Citations should be preceding punctuation.

Reference numbers have been presented in square brackets throughout the manuscript.

3) Please cite your Supporting Information as outlined here: <https://eur04.safelinks.protection.outlook.com/?url=https%3A%2F%2Fjournals.plos.org%2Fplosmedicine%2Fs%2Fsupporting-information&data=05%7C02%7Cf.t.bosch%40amsterdamumc.nl%7C5e27a0a170e84af1116d08dc11291a41%7C68dfab1a11bb4cc6beb528d756984fb6%7C0%7C0%7C638404115437335180%7CUnknown%7CTWFpbGZsb3d8eyJWIjoiMC4wLjAwMDAiLCJQIjoiV2luMzIiLCJBTiI6Ik1haWwiLCJXVCI6Mn0%3D%7C3000%7C%7C%7C&sdata=5MQDTyBBeADLaQMTfGubUcXdZsdWF1QU1qjzUwbAdd8%3D&reserved=0>

Citing of supporting information has been adjusted throughout the manuscript according to the journal guidelines in above link.

FINANCIAL DISCLOSURE

Please enter a financial disclosure statement that describes the sources of funding for the work included in this submission. If the study received no specific funding, please enter "The author(s) received no specific funding for this work."

The following text has been added to page 14:

*“Financial disclosure*

*The Department of Clinical Epidemiology receives funding for other studies from companies in the form of research grants to (and administered by) Aarhus University. The author(s) received no specific funding for this work.”*

COMPETING INTEREST

All authors must declare their relevant competing interests per the PLOS policy, which can be seen here: <https://eur04.safelinks.protection.outlook.com/?url=https%3A%2F%2Fjournals.plos.org%2Fplosmedicine%2Fs%2Fcompeting-interests&data=05%7C02%7Cf.t.bosch%40amsterdamumc.nl%7C5e27a0a170e84af1116d08dc11291a41%7C68dfab1a11bb4cc6beb528d756984fb6%7C0%7C0%7C638404115437335180%7CUnknown%7CTWFpbGZsb3d8eyJWIjoiMC4wLjAwMDAiLCJQIjoiV2luMzIiLCJBTiI6Ik1haWwiLCJXVCI6Mn0%3D%7C3000%7C%7C%7C&sdata=vLWCX7b1%2Fo%2FPj%2FQWIf8xUdWylvkAdCrsI0eMMyMvvDk%3D&reserved=0>

Potential competing interests have been reported on page 14.

Please add this statement to the manuscript's Competing Interests: "SC is an Academic Editor on PLOS Medicine's editorial board."

TITLE

Please include the study setting, i.e. Denmark, in the title.

We changed the title to: ‘Direct factor Xa inhibitors and the risk of cancer and cancer mortality: a Danish population-based cohort study in Denmark’

ABSTRACT

1) PLOS Medicine requests that main results are quantified with 95% CIs as well as p values. When reporting p values please report as p<0.001 and where higher as the exact p value p=0.002, for example. For the purposes of transparent data reporting, if not including the aforementioned please clearly state the reasons why not.

Based on the current recommendations on p-values and statistical significance (reference: https://www.nature.com/articles/d41586-019-00857-9), researchers are advised to avoid using p-values when describing the results of regression models and concluding about significant associations. We believe that reporting of point estimates with corresponding confidence intervals allows for better understanding of strengths and precision of associations. We have added p-values to Table 2, however we would prefer to avoid presenting them in the text.

2) Throughout, suggest reporting statistical information as follows to improve clarity for the reader "22% (95% CI [13%,28%]; p</=)". Please amend throughout the abstract and main manuscript. Please note the use of commas to separate upper and lower bounds, as opposed to hyphens as these can be confused with reporting of negative values.

Statistical information has been reported as suggested.

3) When a p value is given, please specify the statistical test used to determine it.

Chi-square tests have been used to determine P-values. This has been added to the legend of Table 2.

4) Please structure your abstract using the PLOS Medicine headings (Background, Methods and Findings, Conclusions). Please combine the Methods and Findings sections into one section, "Methods and findings".

The abstract has been changed according to journal guidelines

5) Please explain what rivaroxaban is, e.g. "...rivaroxaban, a direct anticoagulant and factor Xa inhibitor,.." or similar.

The introduction of the abstract has been changed to *“rivaroxaban, a direct anticoagulant and factor Xa inhibitor”*

6) Please ensure that all numbers presented in the abstract are present and identical to numbers presented in the main manuscript text.

All numbers presented in the abstract are now identical to numbers presented in the main manuscript text.

7) Please include the age of the study population and the main outcome measures.

The mean age in both groups and main outcome measures are now included in the abstract.

8) Please include the important dependent variables that are adjusted for in the analyses.

The following sentence on variables included in the propensity score model has been added to the abstract: *“Propensity scores were estimated using logistic regression and including in the model sex, age group at index date, comorbidities, and use of co-medications.”*

9) In the last sentence of the Abstract Methods and Findings section, please describe the main limitation(s) of the study's methodology.

We have added the following sentence on limitations to the abstract: *“The main limitation with this method is the possibility of residual confounding.”*

AUTHOR SUMMARY

At this stage, we ask that you include a short, non-technical Author Summary of your research to make findings accessible to a wide audience that includes both scientists and non-scientists. The Author Summary should immediately follow the Abstract in your revised manuscript. This text is subject to editorial change and should be distinct from the scientific abstract. Please see our author guidelines for more information: <https://eur04.safelinks.protection.outlook.com/?url=https%3A%2F%2Fjournals.plos.org%2Fplosmedicine%2Fs%2Frevising-your-manuscript%23loc-author-summary&data=05%7C02%7Cf.t.bosch%40amsterdamumc.nl%7C5e27a0a170e84af1116d08dc11291a41%7C68dfab1a11bb4cc6beb528d756984fb6%7C0%7C0%7C638404115437335180%7CUnknown%7CTWFpbGZsb3d8eyJWIjoiMC4wLjAwMDAiLCJQIjoiV2luMzIiLCJBTiI6Ik1haWwiLCJXVCI6Mn0%3D%7C3000%7C%7C%7C&sdata=lJiOvUz2k3mHWw62JsbZAAD8FsGsGjOgMuzzxcjs004%3D&reserved=0>.

The summary should include 2-3 single sentence, individual bullet points under each of the questions. The last bullet under 'What Do These Findings Mean?' point should describe the main limitation of the study's methodology.

It may be helpful to review currently published articles for examples which can be found on our website here <https://eur04.safelinks.protection.outlook.com/?url=https%3A%2F%2Fjournals.plos.org%2Fplosmedicine%2F&data=05%7C02%7Cf.t.bosch%40amsterdamumc.nl%7C5e27a0a170e84af1116d08dc11291a41%7C68dfab1a11bb4cc6beb528d756984fb6%7C0%7C0%7C638404115437335180%7CUnknown%7CTWFpbGZsb3d8eyJWIjoiMC4wLjAwMDAiLCJQIjoiV2luMzIiLCJBTiI6Ik1haWwiLCJXVCI6Mn0%3D%7C3000%7C%7C%7C&sdata=NrPp8fhwhuSlwpPvZCYBKBx053AQA3V8JLy9w8%2FMsig%3D&reserved=0>

We added the following author summary below the abstract:

Author summary

Why Was This Study Done?

- Cancer incidence is growing worldwide
- Factor Xa inhibitors might prevent cancer progression and spread

What Did the Researchers Do and Find?

- We assessed cancer incidence during 5 years of follow-up in patients with atrial fibrillation in Denmark using a factor Xa inhibitor or a thrombin inhibitor (dabigatran) for stroke prevention.
- Compared to dabigatran, factor Xa inhibitors were not associated with a reduced risk of cancer during follow-up.

What Do These Findings Mean?

- Factor Xa inhibitor use for atrial fibrillation did not appear to significantly reduce cancer risk compared to dabigatran use.

INTRODUCTION

1) p.2: Please introduce the abbreviation "RCT" before its first use.

RCT has been written in full at first use.

2) "Pharmacological blockade...immune checkpoint inhibition." - please provide reference.

A reference has been added to this sentence on page 3.

METHODS AND RESULTS

General points:

1) For all observational studies, in the manuscript text, please indicate: (1) the specific hypotheses you intended to test, (2) the analytical methods by which you planned to test them, (3) the analyses you actually performed, and (4) when reported analyses differ from those that were planned, transparent explanations for differences that affect the reliability of the study's results. If a reported analysis was performed based on an interesting but unanticipated pattern in the data, please be clear that the analysis was data-driven.

All points mentioned above are presented in the methods section of the manuscript

2) Did your study have a prospective protocol or analysis plan? Please state this (either way) early in the Methods section.

a) If a prospective analysis plan (from your funding proposal, IRB or other ethics committee submission, study protocol, or other planning document written before analyzing the data) was used in designing the study, please include the relevant prospectively written document with your revised manuscript as a Supporting Information file to be published alongside your study, and cite it in the Methods section. A legend for this file should be included at the end of your manuscript.

b) If no such document exists, please make sure that the Methods section transparently describes when analyses were planned, and when/why any data-driven changes to analyses took place.

c) In either case, changes in the analysis-- including those made in response to peer review comments-- should be identified as such in the Methods section of the paper, with rationale.

There was a prospective protocol which will be added to the revised submission. We have added the following sentence at the beginning of the methods section: *“This study was executed according to a prospective protocol, which can be found in the supplemental material (S1 Appendix).”*

3) Please ensure that the study is reported according to the STROBE guideline and include the completed STROBE checklist as Supporting Information. When completing the checklist, please use section and paragraph numbers, rather than page numbers. Please add the following statement, or similar, to the Methods: ""This study is reported as per the Strengthening the Reporting of Observational Studies in Epidemiology (STROBE) guideline (S1 Checklist).""

A STROBE checklist has been added to the revised submission.

4) PLOS Medicine requests that main results are quantified with 95% CIs as well as p values. When reporting p values please report as p<0.001 and where higher as the exact p value p=0.002, for example. For the purposes of transparent data reporting, if not including the aforementioned please clearly state the reasons why not. Please include any important dependent variables that are adjusted for in the analyses. We suggest reporting statistical information as detailed above - see under ABSTRACT.

P-values are added to the tables as mentioned above. We want to propose that we can leave them out of the main text with considerations mentioned above.

5) Please present numerators and denominators for percentages, at least in the Tables [not necessarily each time they're mentioned].

We have added numerators and denominators for percentages in the results section on page 9 and 10.

6) Please define "lost to follow-up" as used in this study. Other reasons for exclusion should be defined.

In Denmark, we have virtually complete follow-up. We have defined “loss to follow-up” based on the Danish Civil Registration System, because in a very few cases we cannot follow the patients until death or study end.

We have added the following section to the methods section: “*There was virtually complete follow-up in all patients, however in very few cases (<10) we cannot follow the patients until death, emigration, or study end.”*

7) p.6: Please define 'NSAID'.

NSAID has been defined.

8) p.7: "The study was reported to the Danish Data Protection Board by Aarhus University" - Please confirm that this specific study was reviewed and approved by an institutional review board (ethics committee) before the study began and provide additional details regarding consent. Please provide the specific name of the ethics committee/IRB that approved your study. Once you have amended this/these statement(s) in the Methods section of the manuscript, please add the same text to the "Ethics Statement" field of the submission form.

According to Danish legislation, registry-based research does not require ethical approval and informed consent but only permission from the Danish Data Protection Board. We have added this to statement and the record number to the manuscript with the following text:

*"According to Danish legislation, registry-based research does not require ethical approval and informed consent but only permission from the Danish Data Protection Board. This study was approved by the Danish Data Protection Agency through registration at Aarhus University (Record Number 2016-051-000001/812).”*

9) p.7: "Of these patients, 11,742 (50%) received a factor Xa inhibitor, and 11,970 received dabigatran (50%; Table 1)." - To make the values closer to the actual distribution (49.5% and 50.5%), we suggest adding a decimal point to the percentages.

We have changed this as suggested.

10) p.7: Please define 'SD' at first use.

SD is now defined at first use.

11) p.7: "were more often female (47.8% vs 41.4%), were less often included in the period between 2011 and 2013 (15.6% vs 61.3%), and had more comorbidities than those in the dabigatran cohort (Table 1)." - please revise.

*“Patients on factor Xa inhibitors”* was added at the beginning to complete the sentence.

12) p.8: "After IPTW, the all-cause mortality was higher in the factor Xa inhibitor cohort than the dabigatran cohort (HR 1.17, 95% CI 1.13-1.21; Table 2 and Figure 2)." - Should HR be SHR here?

Indeed, this should be SHR. It was changed in the manuscript.

DISCUSSION

1) p.9 "A pre-clinical study in mice by Graf et al. has shown that factor Xa is a crucial driver of the innate immune signaling pathway." - please provide the reference number.

A reference number has been added.

2) p.10: Please define 'FX' or write in full.

It was changed to written in full

3) p.10: Please define 'VKA' or write in full.

It was changed to written in full

TABLES

1) Please define abbreviations used in each table (including those in Supporting Information files).

All abbreviations in tables have been defined.

2) Please note the use of commas to separate upper and lower bounds, as opposed to hyphens as these can be confused with reporting of negative values.

3) Table 2: Please define 'IPTW', 'CI', 'HR'.

These abbreviations have been defined.

FIGURES

1) For all Figures, please ensure that you have complied with our figures requirements <https://eur04.safelinks.protection.outlook.com/?url=http%3A%2F%2Fjournals.plos.org%2Fplosmedicine%2Fs%2Ffigures&data=05%7C02%7Cf.t.bosch%40amsterdamumc.nl%7C5e27a0a170e84af1116d08dc11291a41%7C68dfab1a11bb4cc6beb528d756984fb6%7C0%7C0%7C638404115437335180%7CUnknown%7CTWFpbGZsb3d8eyJWIjoiMC4wLjAwMDAiLCJQIjoiV2luMzIiLCJBTiI6Ik1haWwiLCJXVCI6Mn0%3D%7C3000%7C%7C%7C&sdata=4bhgccfoDmT%2BFicvFXsvLEdjrzpBGW%2FV5oQ8PzpdpLo%3D&reserved=0>.

2) Please consider avoiding the use of red and green in order to make your figure more accessible to those with colour blindness.

We have changed the figures to dashed and continuous lines.

3) Please in the figure legend/description, define abbreviations used in each figure (e.g. 'IPTW'; including those in Supporting Information files).

Abbreviations have been added to the figure legends

4) Please provide titles, legends and descriptions for all figures (including those in Supporting Information files).

Titles, legends and descriptions have been provided for all figures, including those supplemental figures.

SUPPLEMENTARY MATERIAL

1) For supplementary figures and tables, please see the general comments under TABLES and FIGURES (color, abbreviations, titles, descriptions, etc.) and amend accordingly.

Figures have been changed accordingly

2) We suggest reporting statistical information as detailed above - see under ABSTRACT. Please define all numerical values (e.g. Supplemental Table 4 does not specify the numerical value of the numbers in brackets).

Statistical information has been homogenized according to the suggestions. 95% CI has been added to the first column in supplemental table 4.

3) Supplemental Figure 1: Please ensure that all covariates and their abbreviations are properly defined as well as the abbreviations used in the figure legend.

All abbreviations in supplemental figure 1 have been added to the figure legend

4) Supplemental Figure 2: Please ensure to reference Supplemental Figure 2 in the main manuscript. Please describe the meaning of the lines and bars and add a unit for 'Percent'.

Supplemental figure 2 is now referenced to in the manuscript on page 8. The following description has been added to the figure: *“Bars represent histogram of propensity scores in the two cohorts (expressed as percentages per 100) and lines represent kernel density plots of propensity scores prior to inverse probability of treatment weighting.”*

REFERENCES

All references have been changed according to Journal guidelines.

1) PLOS uses the numbered citation (citation-sequence) method and first six authors, et al.

2) Please ensure that journal name abbreviations match those found in the National Center for Biotechnology Information (NCBI) databases (<https://eur04.safelinks.protection.outlook.com/?url=http%3A%2F%2Fwww.ncbi.nlm.nih.gov%2Fnlmcatalog%2Fjournals&data=05%7C02%7Cf.t.bosch%40amsterdamumc.nl%7C5e27a0a170e84af1116d08dc11291a41%7C68dfab1a11bb4cc6beb528d756984fb6%7C0%7C0%7C638404115437335180%7CUnknown%7CTWFpbGZsb3d8eyJWIjoiMC4wLjAwMDAiLCJQIjoiV2luMzIiLCJBTiI6Ik1haWwiLCJXVCI6Mn0%3D%7C3000%7C%7C%7C&sdata=l1s84un%2BCjhLCbzc6Xx1Wh2W3Fi7da5vQQPqxGT1LzI%3D&reserved=0>), and are appropriately formatted and capitalised.

3) Where website addresses are cited, please specify the date of access.

4) Please also see <https://eur04.safelinks.protection.outlook.com/?url=https%3A%2F%2Fjournals.plos.org%2Fplosmedicine%2Fs%2Fsubmission-guidelines%23loc-references&data=05%7C02%7Cf.t.bosch%40amsterdamumc.nl%7C5e27a0a170e84af1116d08dc11291a41%7C68dfab1a11bb4cc6beb528d756984fb6%7C0%7C0%7C638404115437335180%7CUnknown%7CTWFpbGZsb3d8eyJWIjoiMC4wLjAwMDAiLCJQIjoiV2luMzIiLCJBTiI6Ik1haWwiLCJXVCI6Mn0%3D%7C3000%7C%7C%7C&sdata=nn4aG54KnOiLdoF5LPO5g62%2FywsIVtvbVmBDQ4vqGBQ%3D&reserved=0> for further details on reference formatting.

Comments from the reviewers:

Reviewer #1: The authors report on an interesting analysis of incident cancer and mortality in patients receiving either dabigatran or factor Xa inhibitors for atrial fibrillation. The study is very well designed and addresses an important knowledge gap. Overall findings are "negative" but of significance and paper is well-written. My issues include:

1. Overall the findings are of correlation, not causation. Therefore the abstract conclusion and other wording in the paper that Factor Xa inhibitor use "might decrease the risk of several cancer types" is inaccurate. At most, it shows an association with reduced risk, not causality. Also, it should be some, not several.

We thank the Reviewer for the kind words. We agree with the reviewer that we cannot providence definite evidence for a causal effect. Therefore, we have now rephrased the conclusion of the abstract and manuscript, as also discussed in the response to the Editor’s comment. Results from the subgroup analyses were removed from the main conclusions in the manuscript. In the Discussion we changed the sentences on the subgroup analyses to:

*“In the present cohort, we found no evidence for an effect of factor Xa inhibitors on overall cancer occurrence. We did find an association with factor Xa inhibitor use and lower incidence of alcohol- and smoking-related cancers, lung cancer, hematological cancer, and gastro-esophageal cancer, suggesting that factor Xa may play a role in cancer progression in specific cancer types. However, these findings on secondary outcomes should be interpreted with caution. Precision in certain cancer-specific estimates was low. As for the main analysis, potential residual confounding precludes strong conclusions about causality. Yet, as patients with factor Xa inhibitors more often had comorbidities, the incidence of alcohol- and smoking-related cancers and lung cancer would likely have been higher in this group instead of lower. Finally, differences in subgroup outcomes might have been mediated by differences in bleeding risk between the cohorts. For example, gastrointestinal bleeding occurs more frequently in patients using dabigatran, thus potentially leading to an earlier diagnosis of gastrointestinal cancer.[28]*

2. Did the authors adjust for mulitple testing?

We decided not to adjust for multiple testing since experts in statistics and epidemiology increasingly recommend against traditional methods to correct for multiple comparison (e.g., the Bonferroni adjustment), arguing that a policy of not adjusting for multiple comparisons is preferable because it will lead to fewer errors of interpretation when the data under evaluation are actual observations in nature rather than random numbers. (https://journals.lww.com/epidem/abstract/1990/01000/no_adjustments_are_needed_for_multiple_comparisons.10.aspx)

3. Why is the significant finding that overall mortality is increased in patients receiving factor Xa inhibitors not reported in the abstract but cancer-specific mortality which had no difference is reported? Both are secondary outcomes per Methods. This subject also deserves a robust discussion.

We agree with the Reviewer that this secondary outcome should be mentioned in the abstract. We added the following to the results section of the abstract: “but all-cause mortality was higher in the factor Xa inhibitor cohort (HR 1.17, 95% CI 1.13–1.21)”.

We elaborated on this in the discussion section on page 9 by adding some discussion. It now states:

*“Although we used IPTW to decrease confounding, the all-cause mortality risk was still higher in the factor Xa cohort than the dabigatran cohort, which may be the result of residual confounding. Guidelines suggest to be cautious when prescribing dabigatran to elderly patients because of concerns of bleeding and myocardial infarction.[35–37] Factor Xa inhibitors are also preferred over dabigatran in patients with chronic kidney disease.[36] Nonetheless, previous observational studies suggest that factor Xa inhibitors do not increase mortality compared to dabigatran.[38] The sensitivity analysis excluding the time period of COVID-19, during which factor Xa use was more prevalent, suggests that this pandemic may also have led to a higher mortality risk in the factor Xa group.”*

Reviewer #2: Statistical review

This paper uses Danish healthcare data to explore whether a pre-clinical hypothesis about Direct factor Xa being linked to cancer translated into inhibitors of it leading to lower incidence of cancer. This study is reported well and uses suitable methods. I had a few comments:

1. Abstract: with the significant results for cancer sub-types highlighted, it would be useful to add in the abstract how many distinct types of cancer were included to highlight potential for multiplicity.

We agree with the Reviewer that this is important information. We added the following sentence to the abstract: *“The primary outcome was any cancer. Secondary Secondary outcomes wereS, cancer-related mortality, and all-cause mortality.”*

2. Study population: What was the justification for the +/- three month window for prescription around diagnosis of AF (in particular allowing it to be 3 months prior to diagnosis)

We have included in- and outpatient AF diagnoses, and especially for the outpatients we do not know exactly when the AF diagnosis occurred, since it is registered sometime between the first and last visit date. To emphasize this, we added the following sentence to the methods section on page 4: *“This 3 month window is used since the atrial fibrillation diagnosis in outpatients is registered somewhere between the first and last visit date. Therefore DOAC treatment could have been started before the diagnosis was registered.”*

3. Study outcomes and follow up: "An intention-to-treat approach was used in the main analyses, in which crossover or DOAC discontinuation was not taken into account." - is the mechanism of action of factor Xa consistent with this analysis (i.e. a one-off treatment for a short time period would have long-term preventative effects)? I see later that an on-treatment analysis is used so I would recommend stating here that sensitivity analyses were done as well.

We agree with the Reviewer that this information is missing. We have now added the following sentence the methods section on page 5: *“Additionally, in several sensitivity analyses, we assessed different outcomes in an on-treatment analysis and a time-varying analysis.”*

4. Statistical analysis/results: presumably age at starting treatment is a big factor that would be associated with cancer diagnosis. The covariate balance plots indicate that 'age_3' is not that well balanced and I wonder if a sensitivity analysis that adjusted for age would be useful to add? I believe this might also explain the higher all-cause mortality rates. It is possible that the Fine and Gray model already adjusted for age, in which case I would make this clearer in the statistical analysis section.

We thank the Reviewer for highlighting this important point, which we addressed at the beginning of the rebuttal in the response to the Editor.

Reviewer #3: This population-based retrospective cohort study aimed to evaluate whether patients with atrial fibrillation on direct factor Xa inhibitors exhibit a reduced risk of cancer and cancer-related mortality compared to those taking the direct thrombin inhibitor dabigatran. The authors found that the use of Factor Xa inhibitors was not associated with a lower overall cancer incidence. Nevertheless, subgroup analyses suggested a potential decrease in the risk of several cancer types. The study is intriguing, and its results hold significant clinical value. The research design, overall, seems robust. I have a few questions and suggestions, as follows:

1. The authors mention, "Patients were followed from the date of the DOAC initiation until ...... or 5 years of follow-up, whichever came first." However, since the development of cancer from inception to diagnosis is generally believed to require a considerable amount of time, I am curious as to why the authors have chosen to limit the follow-up period to 5 years. This duration might be insufficient to observe a new development of cancer.

We thank the Reviewer for highlighting this important point, which we discussed in the response to the Editorial comments above.

2. Please provide the mean follow-up duration. Is the actual average follow-up time less than 5 years? Considering cancer as the outcome, do the authors believe that this duration of follow-up is sufficient?

All patients were followed for five years, including patients included in 2015 (end of inclusion period), Combined with the abovementioned considerations we feel that follow-up time should be sufficient to assess outcomes.

3. The reviewer recommends that the authors clarify their choice of using Fine and Gray competing risk models to estimate the Subdistribution Hazard Ratio (SHR) in the manuscript. Additionally, it would be informative to know whether a cause-specific Cox model could be employed to estimate the Hazard Ratio (HR) as part of a sensitivity analysis.

We preferred a Fine and Gray model in our study because the cumulative incidences and Fine-Gray models have a direct one-to-one correspondence even in presence of competing events. We agree with the Reviewer that a cause specific HR analysis is an interesting addition. We have added this as a sensitivity analysis.

4. The reviewer requests that the authors clearly describe the methods used to construct cumulative incidence curves in the "Statistical Analyses" section of the manuscript.

We have added the following sentence to the statistical analysis section on page 6: *“Using the propensity score weighted cohorts, we constructed cumulative incidence curves for the outcomes of cancer, all-cause mortality, and gastrointestinal bleeding, by calculating the 5-year cumulative incidences for each outcome, comparing the factor Xa inhibitor cohort with patients treated with dabigatran, using the Aalen-Johansen estimator, which accounts for the competing risk of death.”*

5. In the Abstract, the authors clearly present that the use of Factor Xa inhibitors was associated with a lower risk of lung cancer, hematological cancer, and gastro-esophageal cancer. However, these findings are not mentioned in the Discussion summary or conclusion. Could the authors consider including these findings in the summary/conclusion?

We changed the wording of the abstract conclusion according to the remarks made by Reviewer number 1, in which the conclusion of the findings of these subgroups are noted. .

6. Although the main analysis indicates a significantly lower risk of lung cancer, hematological cancer, gastro-esophageal cancer, and smoking- and alcohol-related cancers in Factor Xa inhibitor users, many results from the on-treatment and time-varying analyses (as part of sensitivity analyses) do not achieve statistical significance. What do the authors perceive as the potential reasons for these discrepancies? It would be beneficial to address this in the Discussion section.

We agree with the Reviewer that this should be addressed more clearly in the discussion section. We added additional text to the discussion section on differences between sensitivity analysis.

In the main conclusion at the beginning of the discussion section:

*“Although the upper bound of the 95% CI of the primary analysis was 1.00, suggesting a possible effect, no difference was found in any of the sensitivity analysis, including an analysis in which we excluded the first year of COVID-19 during which fewer cancer diagnosis were made, possibly resulting in fewer cancer incidences in the factor Xa inhibitor group.”*

And further in the discussion section on page 13 (track change document):

*“Finally, not all subgroup analysis showed the same results in sensitivity analysis. The time-varying results might differ from the intention-to-treat and on-treatment analyses, given that in time-varying analyses we have not used the IPTW approach as they were directly adjusted for confounding variables, resulting in decrease in the precision of the estimates. In the on-treatment analysis patients were followed for 200 days after last subscription, resulting in a shorter overall follow-up time. Although the intention-to-treat en on-treatment analyses show largely the same results, shorter follow-up time could have an effect on outcomes.”*

7. Could the observed discrepancies among different cancer types be potentially attributed to a Type 1 error, arising from the issue of multiple comparisons?

This point was discussed above in the second comment by reviewer 1.

8. Is there a potential pathophysiological mechanism that can explain the differing outcomes for various types of cancer

We agree with the reviewer that this could be addressed in the discussion section. We have added the following sentences to the discussion section:

*“Mechanisms of innate immune evasion are different across tumor types and tumors respond differently to immunotherapy. Therefore, based on this hypothesis, differences in response to factor Xa inhibition across tumor types are expected.”*

Reviewer #4: This is an excellent claims based database study. The question is scientifically valid and the approach and methodology solid. The authors have used an intention to treat analysis as the primary approach and included on treatment and time varying covariate analysis for the secondary/sensitivity analysis. The results are well presented and discussed. I do not have any recommendations to improve this manuscript. I congratulate the authors on an excellent, interesting and relevant study.

We thank the reviewer for the kind words.
