# Supplementary material for: Direct factor Xa inhibitors and the risk of cancer and cancer mortality: A Danish population-based cohort study
Source: PLoS Med. 2024 Jul 1;21(7):e1004400. doi: 10.1371/journal.pmed.1004400 (PMC11251598; doi:10.1371/journal.pmed.1004400)
Supplement: Attachment — Submitted filename: Response to the Editor and Reviewers.docx [file pmed.1004400.s013.docx]

ACADEMIC EDITOR COMMENTS

I think the authors have done a reasonable job of responding to the reviewers' comments.

We thank the Academic Editor for the kind comments

I would like to note that this is a completely negative study, and furthermore, the Factor Xa inhibitors increased all-cause mortality compared to standard thrombin inhibitors.

I think the authors need to highlight the increase in all-cause mortality with factor Xa inhibitors in the conclusion of their abstract and in the "What did the researchers do and find?" and "What do these findings mean?" sections of the Author Summary. It is important that this important (and adverse) finding be clearly stated and obvious even to the quick reader.

We thank the Editor for the suggestion. We added the following sentence to the conclusion of the abstract. *“We did observe an increase in all-cause mortality in the factor Xa inhibitor cohort.”*

We changed the author summary to: “*No difference in cancer-related mortality (SHR 1.03, 95% CI [0.94,1.12]) was observed, but all-cause mortality was higher in the factor Xa inhibitor cohort (HR 1.17, 95% CI [1.13,1.21]).”*

EDITORIAL COMMENTS

Please be sure to include absolute numbers of events when reporting results, including in the sensitivity analyses, (e.g., #s of all-cause deaths, cancer-specific deaths, cancer subtypes) for the matched group analysis. In the text, it appears that you are reporting the total N for incident cancers (and deaths), but only for the overall group, not after matching, so the absolute numbers of events used for IPT-weighted cumulative incidence/SHR are not available in the text. Please also include the absolute numbers of events in the relevant tables, such as Table 2 (i.e., add two columns in table 2 to report numbers of events in each treatment group for each outcome listed) as well as Tables S4 and S5.

We have added the absolute numbers of events to the results section, including results of the matched groups. Additionally we have added these numbers to the tables. Since the extra columns affected readability in the tables S4 and S5, we have split these tables in S4 to S7. Table S4 does not have total events mentioned, since the results in the intention-to-treat analyses are the same as in table 2. Additionally, for time-varying analysis we were not able to separate these data in the two treatment cohorts.

ABSTRACT

1) l.41: Please define ‘SD’ at first use.

SD was defined at first use

2) ll.46-47: Please include absolute numbers of events and denominators before percentages: e.g., (n/N; 9.11%, 95%CI [8.61%,9.63%]). Please do the same for lines 48-50 and when you report these data in the Results section (e.g., lines 252-253; 259-260, etc.).

We have adjusted this as stated suggested in abstract and throughout the manuscript.

3) Please state the main limitation at the end of the “Methods and Findings” section. Please also consider modifying this sentence as follows, “The main limitations of the study were the possibility of residual confounding and the short follow up period.”

We changed the methods and findings section as suggested.

AUTHOR SUMMARY

Thank for providing the Author Summary. We feel that the Author Summary in its current form does not provide sufficient detail (please also see the comments provided by the Academic Editor). The Author Summary should consist of 2-3 succinct bullet points under each of the following headings:

• Why Was This Study Done? Authors should reflect on what was known about the topic before the research was published and why the research was needed.

• What Did the Researchers Do and Find? Authors should briefly describe the study design that was used and the study’s major findings. Do include the headline numbers from the study, such as the sample size and key findings.

• What Do These Findings Mean? Authors should reflect on the new knowledge generated by the research and the implications for practice, research, policy, or public health. Authors should also consider how the interpretation of the study’s findings may be affected by the study limitations. In the final bullet point of ‘What Do These Findings Mean?’, please describe the main limitations of the study in non-technical language.

We adjusted the author summary as stated below:

**Author summary**

**Why Was This Study Done?**

- A pre-clinical study in mice with breast cancer and fibrosarcoma showed that factor X dampens antitumor immunity and that factor Xa inhibitor promote tumor immunity
- Whether factor Xa inhibition is associated with decreased cancer incidence and cancer-related mortality in humans is unknown.

**What Did the Researchers Do and Find?**

- We assessed cancer incidence during 5 years of follow-up in patients with atrial fibrillation in Denmark using a factor Xa inhibitor (n=11,742) or a thrombin inhibitor (dabigatran) (n=11,970) for stroke prevention.
- No substantial difference in the cumulative incidence of cancer was observed between the factor Xa inhibitor (9.11%, 95% CI [8.61%,9.63%]) and dabigatran (9.68%, 95% CI [9.14%,10.25%]) groups (SHR 0.94, 95% CI [0.89,1.00].
- No difference in cancer-related mortality (SHR 1.03, 95% CI [0.94,1.12]) was observed, but all-cause mortality was higher in the factor Xa inhibitor cohort (HR 1.17, 95% CI [1.13,1.21]).

**What Do These Findings Mean?**

- Factor Xa inhibitor use for atrial fibrillation did not appear to significantly reduce cancer risk compared to dabigatran use.
- The main limitations of the study were the possibility of residual confounding and the short follow up period.

INTRODUCTION

1) Please note that references should be preceding punctuation. Also, in some instances, e.g. line 80, the references are still in uppercase format. Please revise throughout the main manuscript.

All references are changed according to PLOS Medicine guidelines.

2) ll.90-94: For clarity, we suggest adding “in mice” after “fibrosarcoma”.

This was changed as suggested.

METHODS AND RESULTS

1) Please note that changes in the analysis-- including those made in response to peer review comments-- should be identified as such in the Methods section of the paper, with rationale.

We added the following sentence to the methods section before the two sensitivity analysis which were added after the first peer review round: *“After peer review of the manuscript, we added two sensitivity analysis. We performed an additional sensitivity analysis with follow-up time extended to 9 years to increase exposure time to the study drug.”*

2) Please add the following statement, or similar, to the Methods: "This study is reported as per the Strengthening the Reporting of Observational Studies in Epidemiology (STROBE) guideline (S1 Checklist)."

This sentence was added at the end of the methods section.

3) l.247: Please add “IQR” in the parentheses when reporting the interquartile range. Please revise throughout the main manuscript.

We’ve added “IQR” to the first use l.200 and revised it throughout the manuscript.

4) l.278: “0.75–0.87” – Please replace the hyphen with a comma.

This was changed as suggested.

DISCUSSION

1) ll.298-299: “Whether inhibition of factor X also works in other cancer types is unclear.” – We suggest adding here that it is also unclear whether the inhibition of factor X also works in humans given the study was done in mice.

We altered this sentence in the discussion section to:

*“Whether inhibition of factor X also works in humans or in other cancer types is unclear.”*

2) ll.306-307: Please mention that these results should also be interpreted with caution due to low event numbers (if correct); perhaps at the end of the sentence on precision?

We changed this sentence to: *“Precision in certain cancer-specific estimates was low due to low event numbers”*

3) l.347: Please include the reference to Graf et al. Also, please reiterate that the study was a preclinical study in mice.

This was changed as suggested.

4) We suggest briefly discussing the transferability from animal models to humans given your study hypothesis is based on data stemming from animal studies.

We have added the following sentence to the discussion section: *“Transferability from animal models to humans could also be a problem when testing this hypothesis. While the importance of factor X in cancer progression was proved in pre-clinical mice studies, it is unknown whether factor X and factor Xa inhibition plays a similar role in humans and different cancer types.”*

SUPPLEMENTARY MATERIAL

Thank you for providing the STROBE checklist. Please replace the page numbers with paragraph numbers per section (e.g. "Methods, paragraph 1"), since the page numbers of the final published paper may be different from the page numbers in the current manuscript.

Paragraphs per section were added to the STROBE checklist.

SOCIAL MEDIA

To help us extend the reach of your research, please provide any X (formerly known as Twitter) handle(s) that would be appropriate to tag, including your own, your coauthors’, your institution, funder, or lab. Please respond to this email with any handles you wish to be included when we tweet this paper.

We have no X handles to add for publication.

Comments from Reviewers:

Reviewer #2: Thank you to the authors for addressing my previous comments well. I have no further issues to raise.

Reviewer #3: The authors have addressed my previous comments.

We thank the Reviewers for their comments.

Any attachments provided with reviews can be seen via the following link:

------------------------------------------------------------

General Editorial Requests

1) We ask every co-author listed on the manuscript to fill in a contributing author statement. If any of the co-authors have not filled in the statement, we will remind them to do so when the paper is revised. If all statements are not completed in a timely fashion this could hold up the re-review process. Should there be a problem getting one of your co-authors to fill in a statement we will be in contact. YOU MUST NOT ADD OR REMOVE AUTHORS UNLESS YOU HAVE ALERTED THE EDITOR HANDLING THE MANUSCRIPT TO THE CHANGE AND THEY SPECIFICALLY HAVE AGREED TO IT.

2) Please ensure that the paper adheres to the PLOS Data Availability Policy (see <https://eur04.safelinks.protection.outlook.com/?url=http%3A%2F%2Fjournals.plos.org%2Fplosmedicine%2Fs%2Fdata-availability&data=05%7C02%7Cf.t.bosch%40amsterdamumc.nl%7C7c26493b1498485b502508dc475ede95%7C68dfab1a11bb4cc6beb528d756984fb6%7C0%7C0%7C638463719985109755%7CUnknown%7CTWFpbGZsb3d8eyJWIjoiMC4wLjAwMDAiLCJQIjoiV2luMzIiLCJBTiI6Ik1haWwiLCJXVCI6Mn0%3D%7C0%7C%7C%7C&sdata=xjrxds67D1Rx85F%2BxBQy1oaoR9tZaqYzvlGa7D9wL7I%3D&reserved=0>), which requires that all data underlying the study's findings be provided in a repository or as Supporting Information. For data residing with a third party, authors are required to provide instructions with contact information for obtaining the data. PLOS journals do not allow statements supported by "data not shown" or "unpublished results." For such statements, authors must provide supporting data or cite public sources that include it.

3) Please review your reference list to ensure that it is complete and correct. If you have cited papers that have been retracted, please include the rationale for doing so in the manuscript text, or remove these references and replace them with relevant current references. Any changes to the reference list should be mentioned in the rebuttal letter that accompanies your revised manuscript.
